# Supplementary material for: Spermidine Supplementation Protects the Liver Endothelium from Liver Damage in Mice
Source: Nutrients. 2021 Oct 21;13(11):3700. doi: 10.3390/nu13113700 (PMC8617984; doi:10.3390/nu13113700)
Supplement: Supplementary file 1 [file nutrients-13-03700-s001.zip › nutrients-1408731-SI.pdf]

Chemoprophylaxis with spermidine protects the liver endothelium and ameliorates chronic liver injury in mice

Genís Campreciós; Maria Ruart; Aina Anton; Nuria Suárez-Herrera; Carla Montironi; Celia Martínez; Erica Lafoz; Héctor García-Calderó; Marina Vilaseca; Marta Magaz; Mar Coll; Isabel Graupera; Scott L Friedman; Joan Carles García-Pagán; Virginia Hernández-Gea

SUPPLEMENTARY FIGURES AND TABLES

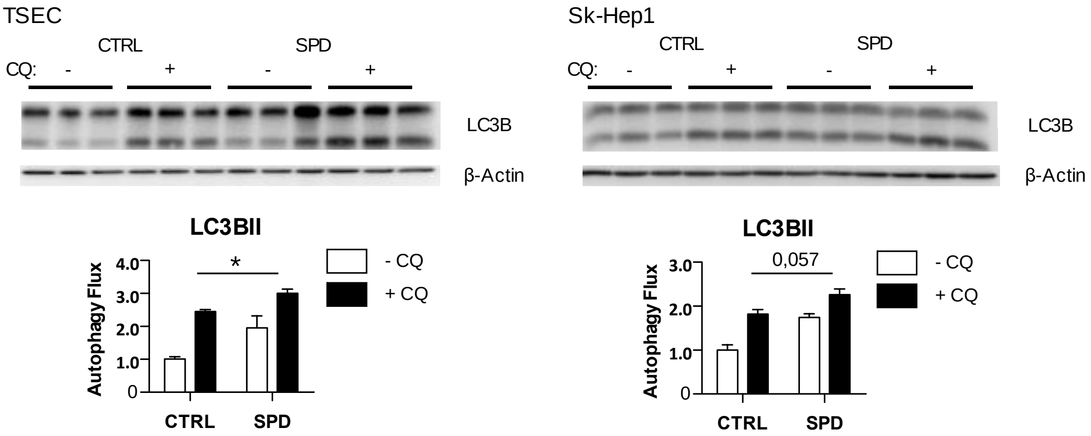

Figure S1. SPD treatment increase autophagy levels in TSEC and Sk-Hep1 cells. TSEC and SK-HEP1 cells were seeded in 6-well plates in triplicates. Next day cells were treated with SPD with or without 20 μM CQ for 24 h. Cells were then collected and analyzed by western blot against LC3B antibody. LC3BII band was quantified and shown in the graphs below. β-Actin was used as loading control. One representative of two independent experiments is shown. Results are the mean ± SEM. *P<0.05 of cells treated with CQ compared to their non-treated controls. Student's t-test.

S2A

CDAAH CDAAH+Spd

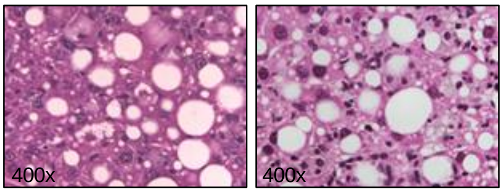

	CDAAH (N=10)	CDAAH +Spd (N=7)
NAFLD ACTIVITY SCORE (0-8)		
0	0	0
1	0	0
2	0	0
3	0	0
4	1	3
5	7	2
6	2	1
7	0	1
8	0	0

	CTRL + CDAAH (N=10)	SPD + CDAAH (N=7)
Steatosis, n		
0	0	0
1	0	0
2	2	1
3	8	6
Ballooning, n		
0	1	4
1	9	3
Lobular inflammation, n		
0	0	0
1	6	4
2	4	1
3	0	2

S2B

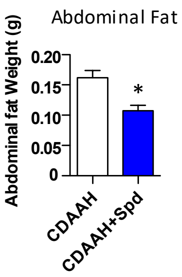

S2C

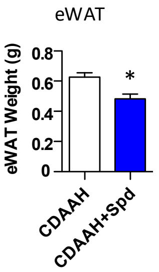

S2D

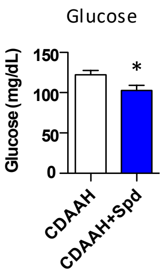

Figure S2. SPD does not modify hepatic steatosis but causes slight improvement in the altered metabolic phenotype in the CDAAH mouse model. A. Representative images and histological liver analysis of steatosis, ballooning and lobular inflammation showing a slight decrease of ballooning without changes in NAFLD ACTIVITY SCORE. B-C. Weight of the abdominal and epididymal adipose tissue, respectively, of mice treated with CDAAH diet with or without SPD supplementation. D Plasma glucose levels in CDAAH mice with or without SPD supplementation. Results are the mean ± SEM. *$P<0.05$ compared to the correspondent control. Exact Fisher test in A and Student's t-test in B-D.

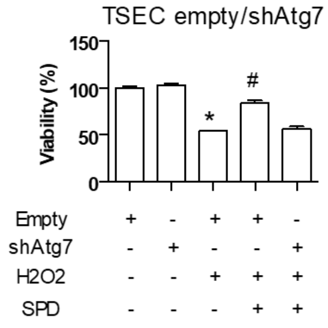

Figure S3. Deficient autophagy impairs SPD protection against oxidative stress in vitro. TSEC infected with an shRNA against Atg7 or with an empty control plasmid were seeded in a 96-well plate in triplicates. Next day cells were pre-treated with SPD for 24 h. Medium was then changed and H2O2 added for another 24 h before adding the MTS solution. One representative of two independent experiments is shown. Results are the mean ± SEM. *P<0.05 compared to the empty control plasmid. #P<0.05 compared to H2O2 treatment. Student's t-test.

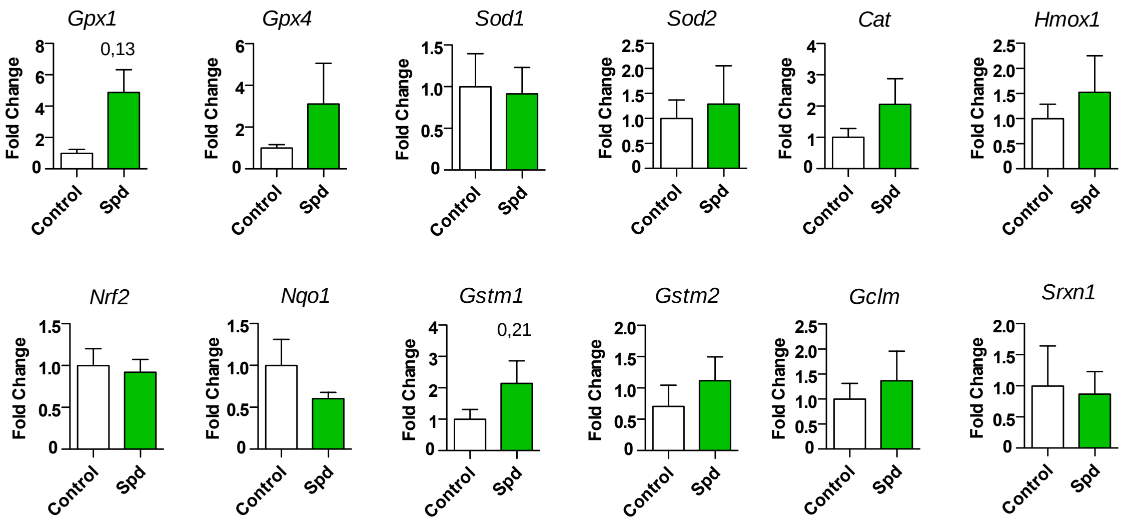

Figure S4. SPD treatment tends to increase classical and NRF2-mediated antioxidant enzyme systems. TSEC were plated in 6-well plates in triplicates. Next day, cells were treated with 10 μM SPD for 24 h and then collected for RNA isolation. Graphs represent the results obtained by qRT-PCR for each indicated gene in controls versus SPD-treated TSEC. Gapdh was used as housekeeping gene. Results are the mean ± SEM. Student's t-test.

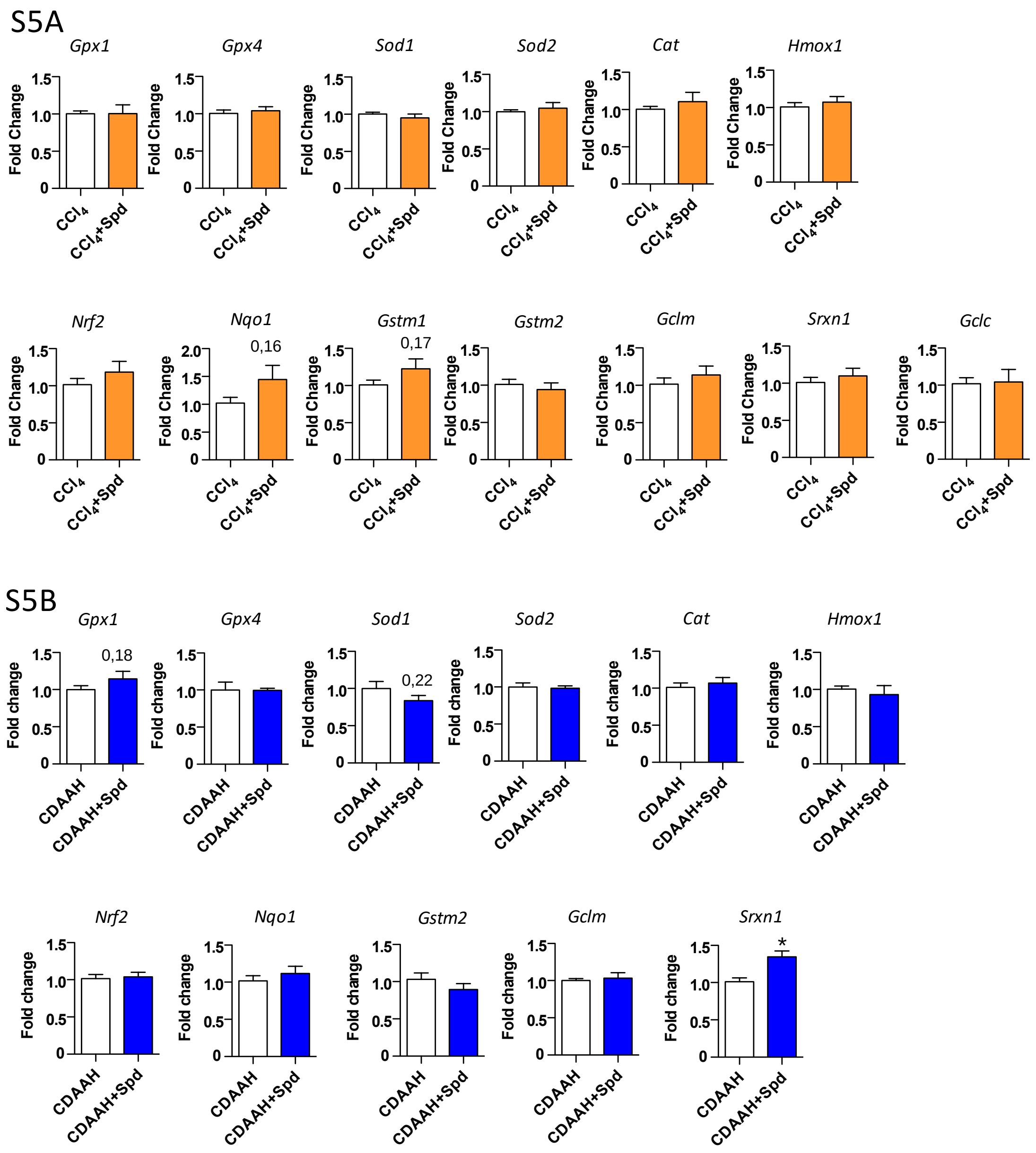

Figure S5. SPD treatment did not show increased hepatic expression of antioxidant enzymes. A-B. qRT-PCR quantification of classical and NRF2-mediated antioxidant enzymes in total liver tissue of mice treated with CCl4 or the CDAAH diet with or without SPD supplementation. Gapdh was used as housekeeping gene. Results are the mean ± SEM. Student's t-test.

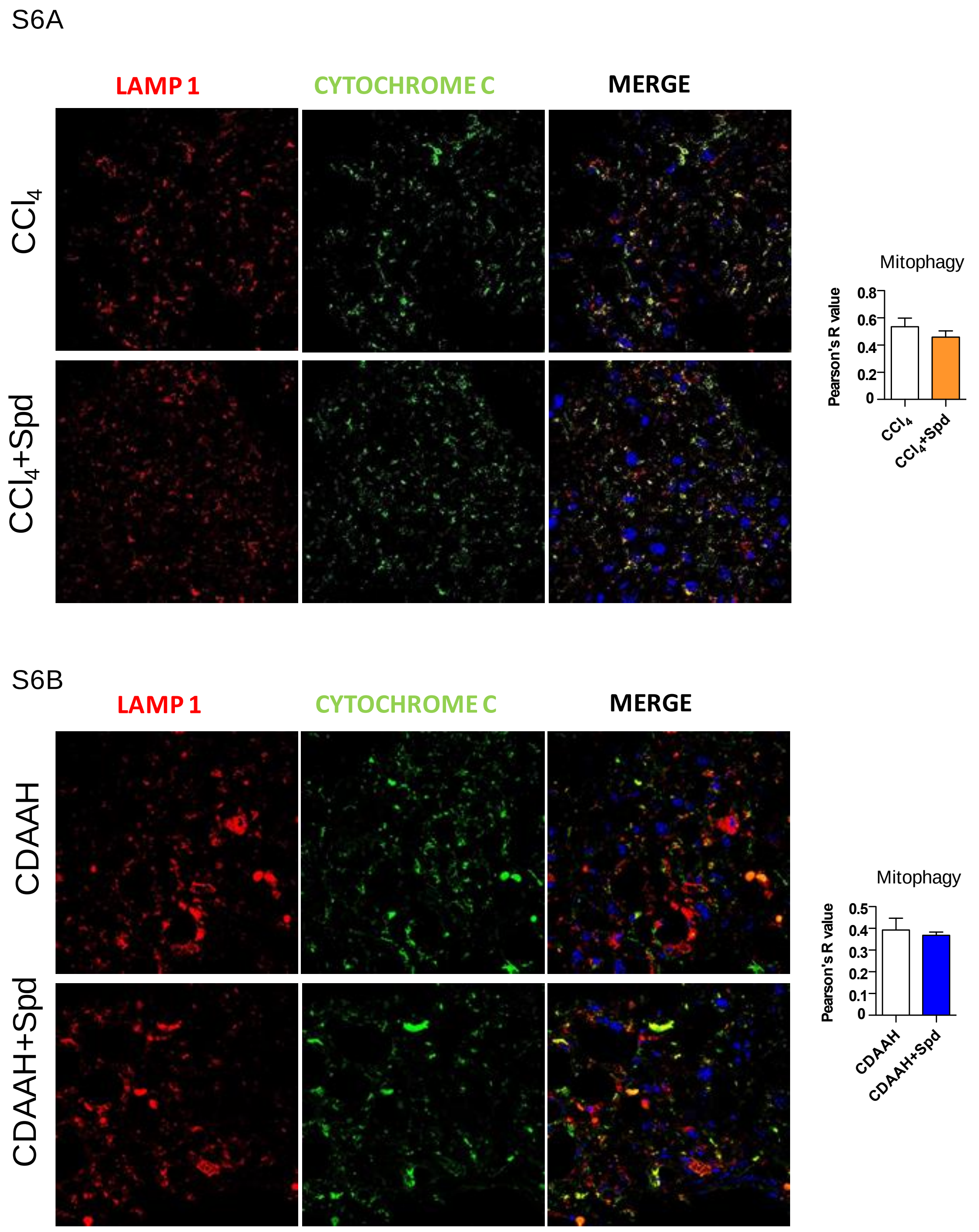

Figure S6. SPD treatment did not show changes in mitophagy activity. A-B. Co-immunofluorescence of LAMP1 and Cytochrome c in FFPE liver sections of mice treated with either CCl4 or the CDAAH diet with or without SPD supplementation. Pearson's correlation between both signals was calculated and shown in the adjacent graphs. Results are the mean ± SEM. Student's t-test.

Supplementary Table S1

Gpx1	**Gpx1_Fw**	**TCGGTTTCCCGTGCAATCA**
	Gpx1_Rv	GTCGGACGTACTTGAGGGAA
Gpx4	Gpx4_Fw	TTACGAATCCTGGCCTTCCC
	Gpx4_Rv	TAGCCGGCTGCAAACTCC
Sod1	Sod1_Fw	CTCACTCTCAGGAGAGCATTCC
	Sod1_Rv	TTCCACCTTTGCCCAAGTCA
Sod2	Sod2_Fw	AAGGAGCAAGGTCGCTTACA
	Sod2_Rv	AATCCCCAGCAGCGGAATAA
Cat	Cat_Fw	GGGATCTTGTGGGAAACAACAC
	Cat_Rv	CTGTGGGTTTCTCTTCTGGCTA
Nrf2	Nrf2_Fw	AGTGGATCCGCCAGCTAC
	Nrf2_ Rv	CTCTGCCAAAAGCTGCATACA
Srxn1	Srxn1_Fw	GCACAACGTACCAATCGCC
	Srxn1_Rv	CAGGGTCCGCCAGGATCG
Nqo1	Nqo1_Fw	TCTCTGGCCGATTCAGAGTG
	Nqo1_Rv	CCAGACGGTTTCCAGACGTT
Gclc	Gclc_Fw	CTGCTGTCCCAAGGCTCG
	Gclc_Rv	TGTACTCCACCTCGTCACCC
Gclm	Gclm_Fw	TGGGCACAGGTAAAACCCAA
	Gclm_Rv	CTGGGCTTCAATGTCAGGGA
Gstm1	Gstm1_Fw	CCGTGCAGACATTGTGGAGA
	Gstm1_Rv	CTGCTTCTCAAAGTCAGGGTTG
Gstm2	Gstm2_Fw	CAGCCCTGACTTTGAGAAAAAGA
	Gstm2_Rv	GACCTTGTTCCCTGCAAACCA
Gapdh	Gapdh_Fw	AGACGGCCGCATCTTCTT
	Gapdh_Rv	TTCACACCGACCTTCACCAT
Actb	Actb_FW	CCCTAAGGCCAACCGTGAAA
	Actb_RV	CAGCCTGGATGGCTACGTAC
